# Immunomodulatory inhibition of osteoclastogenesis by a marine microalgal ethanol fraction targeting T-cells, antigen presentation, and macrophage fate

**DOI:** 10.3389/fimmu.2025.1655321

**Published:** 2025-10-10

**Authors:** Alessio Carletti, Katia Pes, Marco Tarasco, Joana T. Rosa, Sunil Poudel, Hugo Galvão Pereira, Bruno Louro, M. Leonor Cancela, Vincent Laizé, Paulo J. Gavaia

**Affiliations:** ^1^ Centro de Ciências do Mar do Algarve (CCMAR/CIMAR LA), Campus de Gambelas, Universidade do Algarve, Faro, Portugal; ^2^ Faculty of Medicine and Biomedical Sciences, University of Algarve, Faro, Portugal; ^3^ S2AQUA - Collaborative Laboratory, Association for a Sustainable and Smart Aquaculture, Olhão, Portugal; ^4^ GreenCoLab – Associação Oceano Verde, University of Algarve, Faro, Portugal; ^5^ Algarve Biomedical Center, University of Algarve, Faro, Portugal

**Keywords:** osteoimmunology, osteoporosis, T-cells, antigen presentation, macrophages, osteoclast differentiation, microalgae, *Skeletonema costatum*

## Abstract

**Background:**

Targeting immune pathways to prevent bone loss represents a promising, yet underexplored therapeutic strategy.

**Methods:**

An ethanol-soluble fraction derived from the freeze-dried biomass of the marine microalga *Skeletonema costatum* (SKLT) was tested for its ability to modulate immune responses and inhibit osteoclastogenesis. Its effects were evaluated in a zebrafish model of bone regeneration, a medaka model of RANKL-induced osteoporosis, and *in vitro* using murine RAW 264.7 macrophages. Transcriptomic profiling of regenerating fin blastemas at 24 hours post-amputation was performed to identify the affected molecular pathways.

**Results:**

In zebrafish, SKLT treatment suppressed the recruitment of osteoclast precursors and altered mineralization dynamics. Transcriptomic profiling revealed downregulation of genes involved in inflammation, antigen presentation, T-cell activation, and macrophage commitment towards osteoclastogenesis, accompanied by reduced expression of chemokines and cytokines that promote osteoclast precursor recruitment and fusion. In medaka, SKLT significantly reduced vertebral bone loss and enhanced neural arch mineralization in larvae with high RANKL expression. *In vitro*, SKLT inhibited proliferation and osteoclastic differentiation of murine RAW 264.7 macrophages exposed to RANKL without inducing cytotoxicity.

**Conclusion:**

These findings identify *S. costatum* as a source of bioactive immunomodulatory compounds capable of interfering with key osteoimmune mechanisms. Beyond providing proof of concept for their therapeutic potential in bone erosive disorders, this work opens avenues for isolating and characterizing the active molecules, optimizing their delivery, and evaluating their efficacy in preclinical mammalian models. Such strategies could expand the repertoire of safe, nutraceutical-based or adjuvant therapies for osteoporosis and other inflammation-driven skeletal diseases, complementing and potentially enhancing current antiresorptive and anabolic treatments.

## Introduction

Bone erosive pathologies such as osteoporosis represent urgent medical challenges requiring novel therapeutic approaches (1, 2). These disorders arise from the disruption of the equilibrium between bone formation and resorption, ultimately resulting in mineral loss and deterioration of bone structure (3, 4). Current therapeutic strategies rely on a limited number of pharmaceutical agents that either stimulate osteoblast-mediated bone formation (i.e. osteoanabolic compounds) or inhibit osteoclast-driven bone resorption (i.e. antiresorptive drugs). Examples of osteoanabolic drugs are parathyroid hormone analogs (e.g., teriparatide and abaloparatide) (5–7), whereas antiresorptive agents include bisphosphonates and RANKL-monoclonal antibodies (e.g., Denosumab) (8–10). More recently, dual-action drugs such as the anti-sclerostin antibody Romosozumab have emerged as promising options for treating bone loss (11).

Although these drugs demonstrate short-term efficacy, they are not without limitations: their effectiveness diminishes over time (12), and they carry the risk of adverse events—ranging from rare complications such as osteonecrosis of the jaw for antiresorptives (13, 14), to potential associations with osteosarcoma for anabolic agents (5–7).

This therapeutic gap has fueled demand for novel approaches that offer long-lasting, safer alternatives. Accordingly, research has intensified into the mechanisms driving bone erosive diseases, as well as the identification of novel osteoactive compounds with improved safety.

A growing body of research underscores the essential role of the immune system in regulating bone metabolism, a concept that has crystallized into the field of osteoimmunology (15).

Immune cells, particularly macrophages and T-cells, exert critical regulatory control over bone homeostasis (16, 17). Macrophages share a common monocytic lineage with osteoclasts (18), and represent a conserved pool of osteoclasts progenitors in many vertebrates, including fish, mice and humans (19, 20). Together with macrophages, T-cells are the main cellular regulators of inflammation, which is a potent inducer of bone resorption. Inflammation is required for the receptor activator of the nuclear factor kappa-Β ligand (RANKL) signaling pathway, the central molecular driver of osteoclast differentiation (21–25). Chronic inflammation is widely recognized as a key root cause of bone loss in various erosive pathologies (26). For example, postmenopausal osteoporosis is increasingly acknowledged as an inflammatory disease and was (re)named immunoporosis (27–30), where activated T-cells establish chronically increased bone resorption by stimulating osteoclast differentiation through RANKL-dependent and RANKL-independent pathways (31, 32).

The leading role of the immune system in the pathophysiology of osteoporosis and other bone disorders raises the question of whether immunomodulatory therapies could effectively prevent bone loss in patients suffering from such disorders. Surprisingly, few investigations have focused on the potential of targeting immune cells to control bone loss.

The search for novel therapeutic approaches has recently refreshed the role of natural compounds, and marine-derived molecules have become promising assets in drug discovery (33, 34).

This is particularly true for metabolic bone disorders, as an increasing number of studies have identified molecules and extracts obtained from marine organisms with immunomodulatory and osteoprotective bioactivities (35). Among those, microalgae are attracting significant interest owing to a combination of their capacity to synthesize bioactive compounds, technologically advanced cultivation technologies (36–38), and amenability to genetic engineering (39, 40).

Among the recently emerging microalgal species, *Skeletonema costatum*, a marine diatom commonly cultivated in Europe and approved for human consumption (41), has shown promising potential as a source of compounds with biological activity. In detail, the ethanol-soluble compounds extracted from dry biomass of this microalga were characterized by a potent anti-inflammatory activity emerged from *in vitro* studies (42, 43). In the mentioned works, authors performed chemical profiling of ethanolic extract from *Skeletonema* spp. and found them rich in polyunsaturated fatty acids, saturated fatty acids, monounsaturated fatty acids, long-chain aliphatic hydrocarbons, long-chain alcohols, esters, ethers and sterols. Additionally, in a recent work published from our laboratory, ethanolic extract of *S. costatum* had a pro-osteogenic effect in zebrafish larvae and juveniles, as well as fish osteoblastic cell lines (44).

The aim of this study was to evaluate the immunomodulatory and anti-osteoclastogenic potential of the ethanol-soluble fraction of *S. costatum*, and investigate the underlying molecular mechanisms via tissue-specific transcriptomics. To assess the fraction’s osteoactivity, a zebrafish model of bone regeneration (45), a medaka genetic model of RANKL-induced osteoporosis (46), a murine macrophage cell line, were implemented.

## Results

### Skeletonema suppresses osteoclast recruitment and alters bone regeneration dynamics in zebrafish

**Figure 1:**
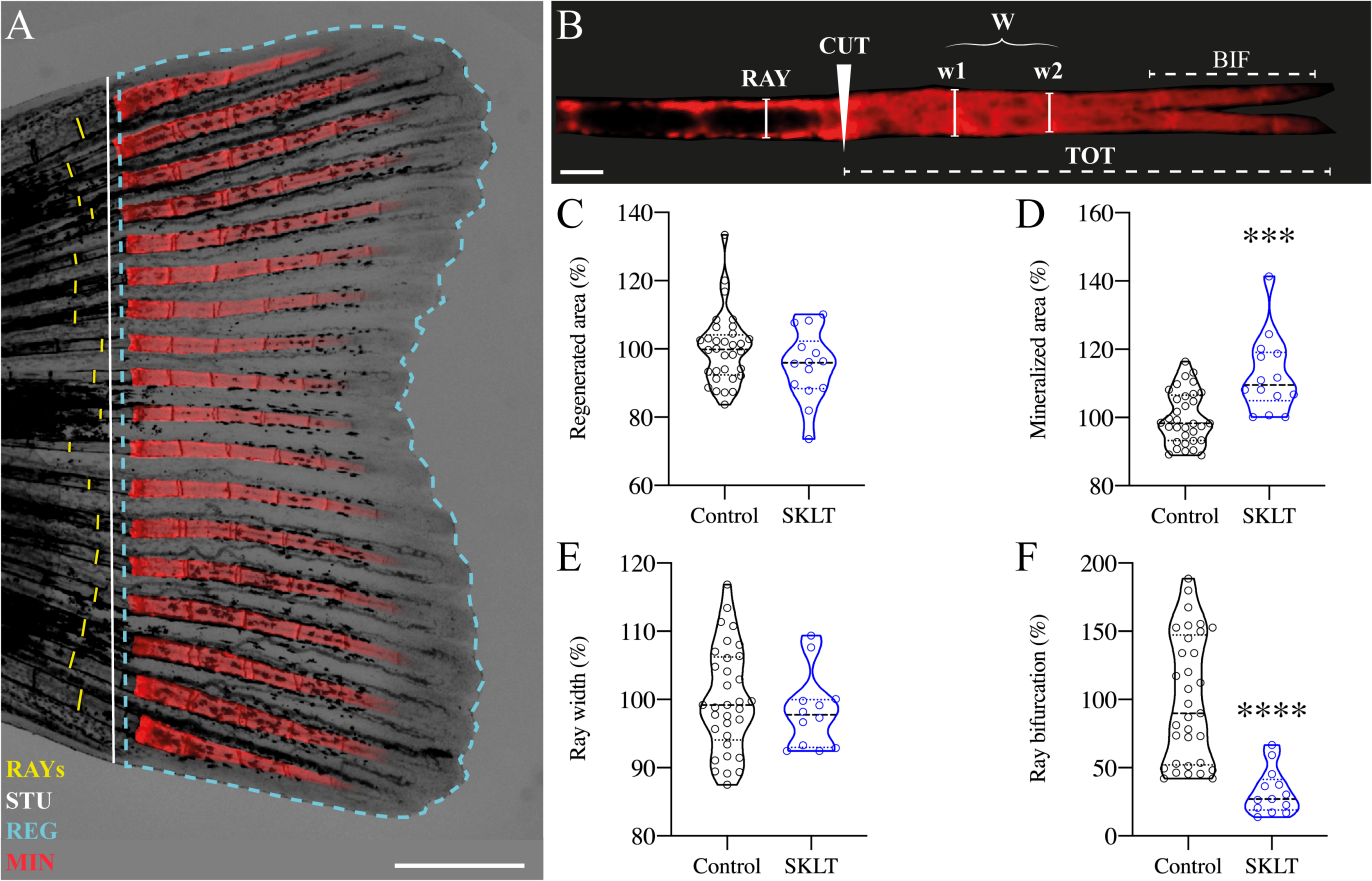
SKLT shifts the mineral equilibrium of regenerating rays toward pro-mineralogenic outcomes. Caudal fin regeneration and mineralization were assessed in young adults exposed to the ethanolic fraction of *S. costatum* (SKLT) or 0.1% ethanol (control). **(A)** Representative image of a caudal fin illustrating the morphometric measurements used to assess the regenerative and mineralogenic effects of SKLT, i.e., ray width (RAY), stump length (STU), regenerated area (REG) and mineralized area (MIN). **(B)** Representative image of an alizarin red S (AR-S) stained fin ray illustrating the morphometric measurements used to assess the patterning effect of SKLT, i.e., amputation plan (CUT), ray width before amputation (RAY), average ray width calculated as the average width of the first two segments after the amputation plan (W), length of bifurcation (BIF) and total length of regenerated ray (TOT). **(C–F)** Effects of SKLT on the regenerated area **(C)**, mineralized area **(D)**, average ray width **(E)** and ray bifurcation **(F)**. Normality was tested through an Anderson–Darling test (*p* < 0.05). Statistical differences were tested through an unpaired *t* test (*p* < 0.05) or a nonparametric Mann–Whitney test (*p* < 0.05) whenever the data distribution resulted non-normal. Asterisks indicate values significantly different at *p* < 0.0005 (***) and *p* < 0.0001 (****). Scale bars are 1 mm in **(A)** and 250 µm in **(B)**.

The impact of the ethanolic fraction of *Skeletonema costatum* (hereafter referred to as SKLT) on bone regeneration was initially evaluated using the zebrafish caudal fin regeneration assay. This model was selected due to its simplicity, high reproducibility, and suitability for studying the coordinated actions of multiple cell types during *de novo* bone formation. It also allows for precise *in vivo* cell tracking (45, 47, 48). Following caudal fin amputation, adult zebrafish were exposed to SKLT by immersion in water supplemented with the fraction. Regenerative outgrowth, mineralization, and morphological patterning of the regenerating bony rays were assessed at a single time point: 5 days post-amputation (5 dpa, **Figures 1A, B**). Quantitative analysis showed a trend toward reduced regenerative area in SKLT-treated animals, though this reduction did not reach statistical significance (**Figure 1C**). In contrast, SKLT exposure resulted in a significant increase in the area of mineralized tissue (**Figure 1D**), suggesting enhanced mineral deposition. While the width of regenerated rays remained unchanged (**Figure 1E**), a marked distalization of ray bifurcation points along the proximal–distal axis was observed in SKLT-treated fish (**Figure 1F**).

**Figure 2:**
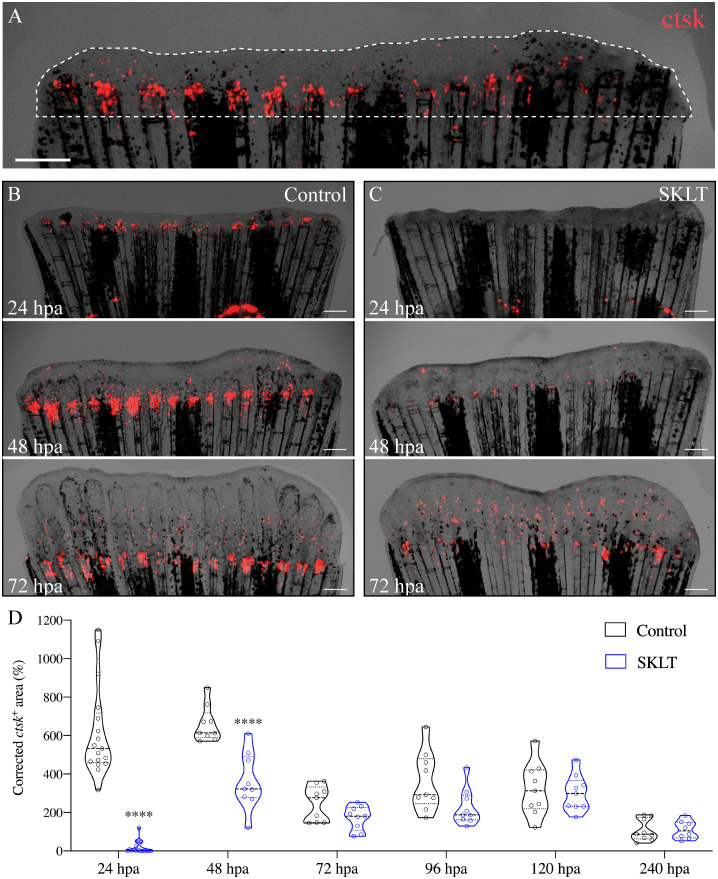
SKLT inhibits the recruitment of osteoclast precursors to the caudal fin blastema. Osteoclastic recruitment was determined from DsRed fluorescence signals observed in the regenerating caudal fin of Tg(*ctsk*:DsRed) transgenic fish. **(A)** Representative image illustrating the regenerated area (dotted line) and *ctsk^+^* area (ctsk, red signal). Time-course of the *ctsk* ^+^ area in regenerating fins of SKLT-treated **(B)** and control fish **(C)**. **(D)** Quantification of the *ctsk* ^+^ area in regenerating fins of SKLT-treated and control fish from 24 to 240 hours post-amputation (hpa). At each timepoint, significant differences were tested through an unpaired *t* test (*p* < 0.05) or a non-parametric Mann-Whitney test (*p* < 0.05) whenever the data distribution resulted non-normal. Asterisks indicate values significantly different at *p* < 0.0001 (****). Scale bars are 500 µm.

To investigate whether the observed effects on mineralization were associated with antiresorptive activity, the dynamics of osteoclast precursors—identified as rounded cathepsin K (*ctsk*)-expressing cells – in Tg(Ola.ctsk:FRT-DsRed-FRT-Cre, *myl7*:EGFP)^mh201^ transgenic zebrafish (49), hereafter referred to as Tg(*ctsk*:DsRed), was monitored in fish continuously exposed to SKLT for 10 days following caudal fin amputation (**Figure 2A**).

In vehicle-treated controls, the number of *ctsk^+^* cells peaked between 24- and 48 hours post-amputation (hpa) (**Figures 2B, D**), followed by a progressive decline in cell density within the regenerated area. In contrast, SKLT-treated fish showed significantly fewer *ctsk^+^* cells recruited to the blastema at both 24 and 48 hpa (**Figures 2C, D**). A delayed peak was observed at 72 hpa, after which *ctsk^+^* cell counts were comparable between SKLT-treated and control animals.

Notably, elongated, tubular *ctsk*-expressing osteoclasts resembling osteolytic tubules (OLTs), previously described in regenerating fin tissue (48), appeared adjacent to the regenerated rays by 96 hpa in control fish, but were delayed until 120 hpa in SKLT-treated fish (**Supplementary Figure S1**).

**Figure 3:**
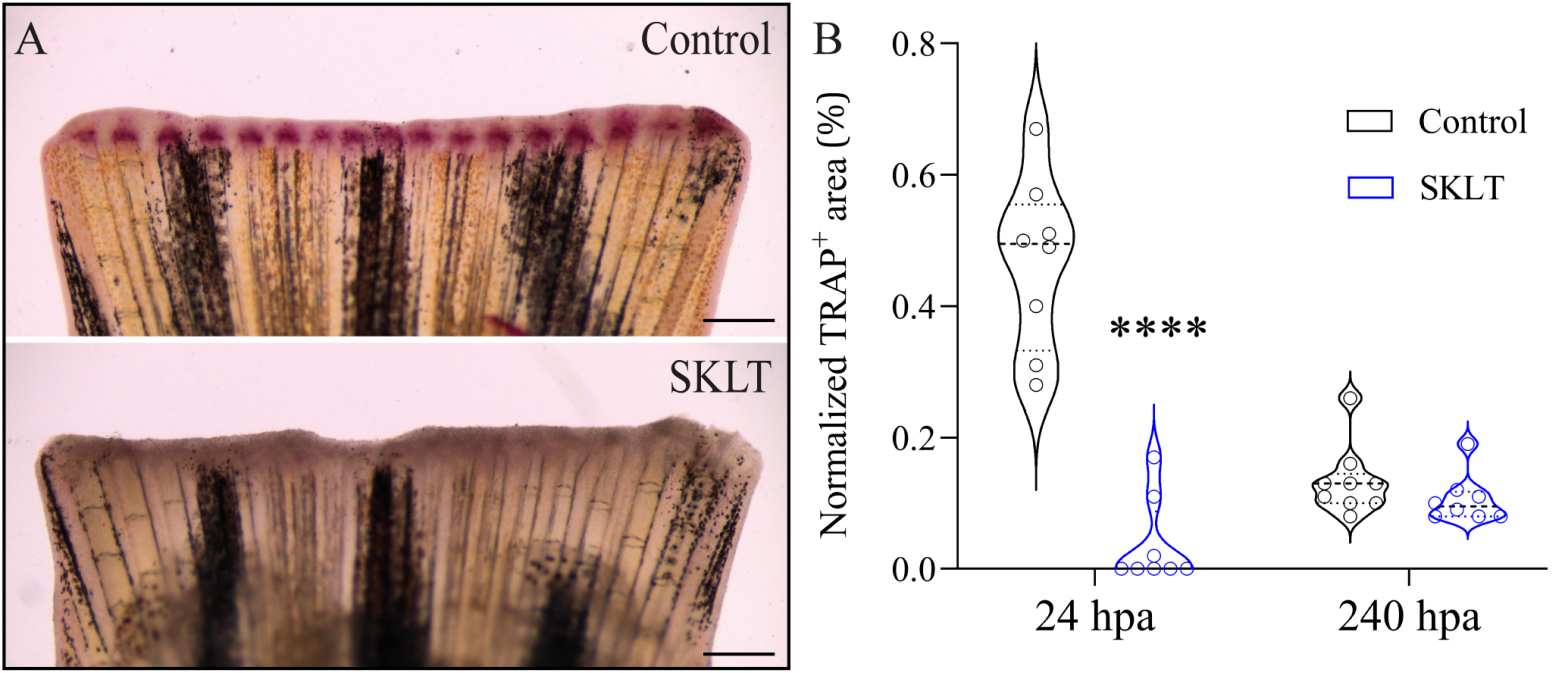
SKLT suppresses bone resorption activity at 24 hours post-amputation. Osteoclast activity was assessed through TRAP (tartrate-resistant acid phosphatase) staining of the regenerating caudal fin of adult zebrafish exposed to the ethanolic fraction of *S. costatum* (SKLT) or 0.1% ethanol (control) at 24 and 240 hours post-amputation (hpa). **(A)** Representative images showing TRAP staining at 24 hpa in control and SKLT-treated fish. **(B)** Quantification of TRAP^+^ area at the early regeneration (24 hpa) and late regeneration (240 hpa) stages. Statistical differences were tested through an unpaired *t* test (*p* < 0.05). Asterisks indicate values significantly different at *p* < 0.0001 (****). Scale bars are 500 µm.

**Figure 4:**
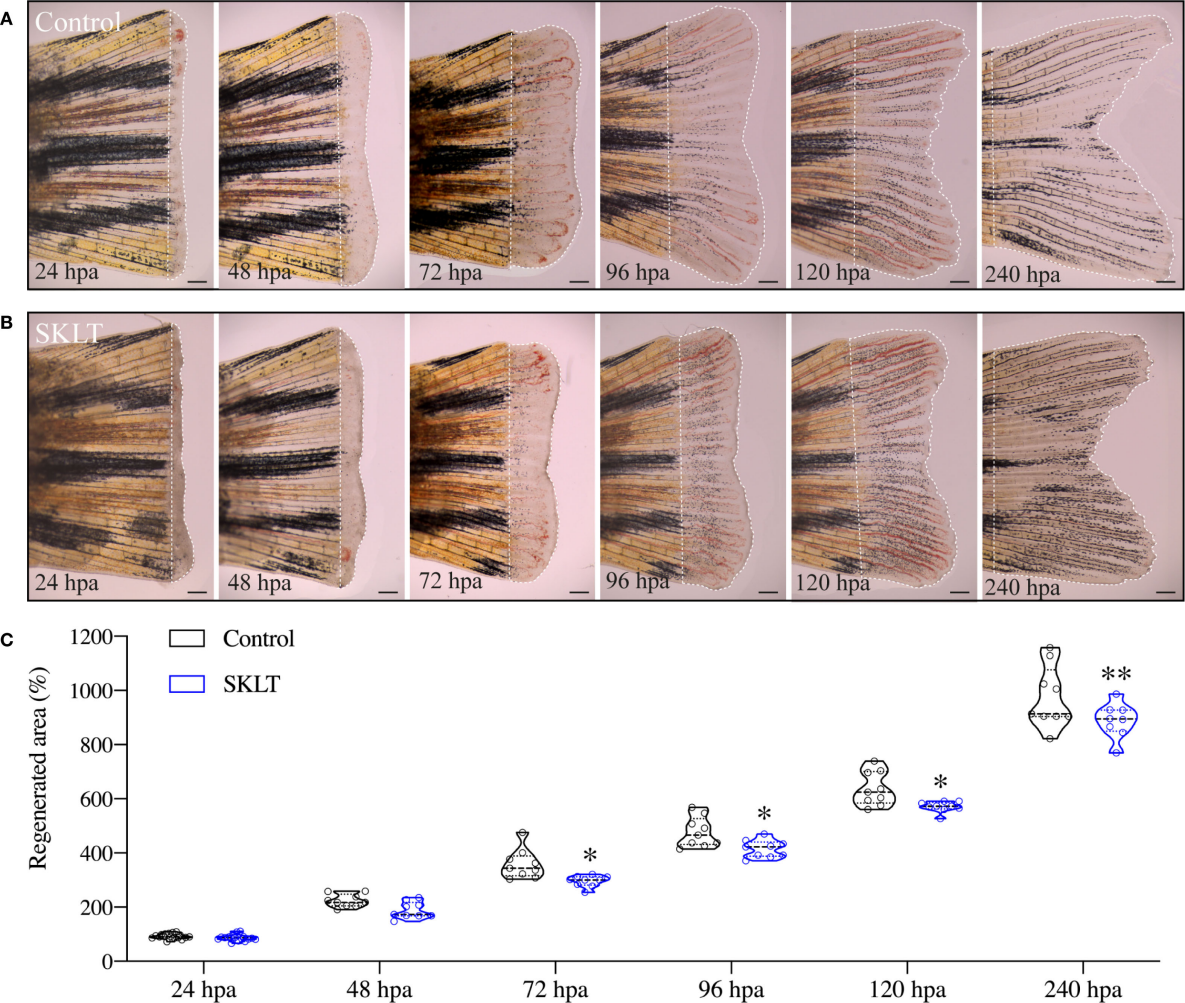
SKLT reduces the overall regenerative performance. Regeneration of the caudal fin was assessed in adult zebrafish exposed to the ethanolic fraction of *S. costatum* (SKLT) or 0.1% ethanol (control) from 24 to 240 hours post amputation (hpa). **(A, B)** Representative images depicting the time-course of caudal fin regeneration (dotted line indicates regenerate) in control **(A)** and SKLT-treated **(B)** fish. **(C)** Quantification of the regenerated area at relevant time points. Statistical differences were tested through an unpaired *t* test (*p* < 0.05) or a non-parametric Mann-Whitney test (*p* < 0.05) whenever the data distribution resulted non-normal. Asterisks indicate values significantly different at *p* < 0.05 (*), *p* < 0.01 (**). Scale bars are 500 µm. Picture illumination was artificially adjusted for better visualization.

This reduction in osteoclast precursor recruitment correlated with a significant decline in early osteoclastic activity. TRAP (tartrate-resistant acid phosphatase) staining, a marker of mature osteoclast function, was nearly absent at 24 hpa in SKLT-exposed animals but returned to levels comparable to controls by 240 hpa (**Figures 3A, B**). Furthermore, in the 10-day exposure experiment, SKLT-treated fish exhibited a sustained reduction in the area of regenerative outgrowth beginning at 72 hpa and persisting through 240 hpa (**Figure 4**). This suggests that the ethanolic fraction may interfere with molecular pathways governing blastema expansion during the regeneration process.

### SKLT suppresses inflammation, T-cell activation, and antigen presentation in the regenerating fin blastema

To investigate whether immunomodulatory mechanism underlies the effects observed in SKLT-treated zebrafish, tissue-specific transcriptomic analysis was performed on regenerating caudal fin blastemas collected at 24 hours post-amputation — the time point at which SKLT exerted the strongest suppression of osteoclast precursor recruitment.

**Figure 5:**
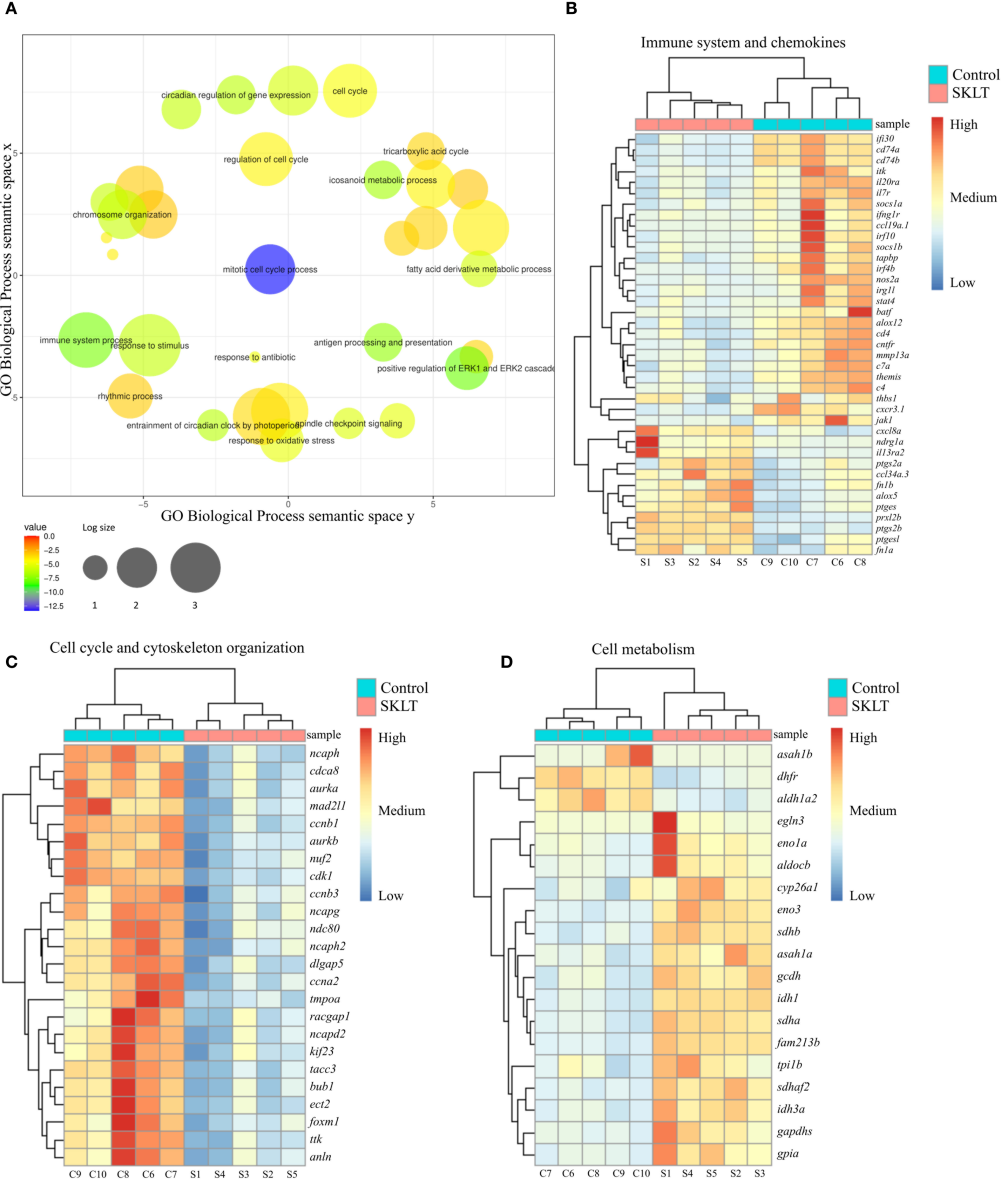
Analysis of the transcriptome of regenerating caudal fin blastema revealed that SKLT modulates immune response, cell cycle and metabolism. RNA-Seq analysis identified differentially expressed genes (DEGs) and enriched gene ontology terms for caudal fin blastemas at 24 hours post-amputation (hpa) in control and SKLT-exposed fish. **(A)** REVIGO plot of biological processes filtered for redundancy and dispensability. Cluster analysis and heatmap of differentially expressed genes associated with **(B)** immune system (GO:0002376), eicosanoid metabolism (GO:0006690), inflammatory response (GO:0006954), T-cell activation (GO:0042110), and cytokine-mediated signaling pathway (GO:0019221). **(C)** Cell cycle (GO:0022402) and cytoskeleton organization (GO:0007010); **(D)** glycolysis (GO:0006096), fatty acid metabolism (GO:0006099), tricarboxylic acid cycle (GO:0006631), and carboxylic acid metabolic process (GO:0019752).

Bulk RNA sequencing identified 720 differentially expressed genes (false discovery rate, FDR< 0.01), of which 361 were upregulated and 359 were downregulated (**Supplementary Figure S2**). Gene ontology (GO) analysis was performed using the DAVID platform, and significantly enriched Biological Process (BP) terms were filtered for uniqueness and plotted using REVIGO (**Figure 5A**). Heatmaps were then generated to visualize the expression patterns of genes associated with the most relevant enriched processes.

Among the most significantly affected biological processes were those associated with both innate and adaptive immune responses. Genes involved in inflammatory signaling—including *irg1l*, *nos2a*, *thbs1*, and *il20ra*—as well as those linked to the interferon system (*ifi30*, *ifng1r*, *irf4b*, *irf10*) and the complement cascade (*c4*, *c7a*) were consistently downregulated in SKLT-treated blastemas (**Figure 5B**). Since interferon gamma (IFN-γ), primarily produced by Th1 cells (50), promotes antigen-dependent T-cell activation and the expression of pro-osteoclastogenic cytokines (50), genes involved in T-cell signaling and antigen presentation were examined. Indeed, multiple genes central to T-cell activation and MHC class II antigen presentation—including *socs1a*, *itk*, *il7r*, *batf*, *cd4*, *cd74a*, *cd74*b, and *jak1 (* 30, 31)—were downregulated following SKLT exposure (**Figure 5B**).

Conversely, several immunoregulatory genes such as *fn1a*, *fn1b* (fibrin isoforms), and *il13ra2* (interleukin 13 receptor alpha 2) were upregulated, consistent with a shift toward anti-inflammatory immune signaling. Chemokine-related paracrine communication appeared differentially modulated: genes encoding Ccl34 (*ccl34a.3*) and Cxcl8 (*cxcl8a*) were upregulated, whereas Ccl19 (*ccl19a.1*) and the chemokine receptor Cxcr3 (*cxcr3.1*) were downregulated.

Together, these transcriptomic data support the hypothesis that *S. costatum* induces complex immunomodulatory effects in the regenerating fin blastema, suppressing both inflammation and T-cell–mediated antigen presentation. This shift in immune signaling likely contributes to reduced recruitment and differentiation of osteoclast precursors.

### SKLT inhibits retinoic acid synthesis and cell proliferation while upregulating metabolic gene expression

Cluster analysis of genes involved in cell cycle regulation and cytoskeletal organization revealed that SKLT exposure may suppress cell proliferation in fin blastemas at 24 hpa (**Figure 5C**).

Downregulated genes included those associated with proliferation (*ttk*, *cdca8*, *dlgap5*, *tacc3*, *nuf2*, *ndc80*), mitosis (*bub1*, *ccnb1*, *ccnb3*, *ccna2*, *cdk1*, *tmpoa*, *mad2l1*, *foxm1*), and cytokinesis (*ect2*, *aurka*, *aurkb*, *kif23*, *racgap1*, *anln*), indicating a broad inhibitory effect of SKLT on cell cycle progression. In contrast, transcriptomic analysis revealed increased expression of genes involved in energy metabolism (**Figure 5D**), suggesting a metabolic shift. Glycolysis-related genes—including *eno3*, *eno1a*, *gapdhs*, *aldocb*, *tpi1b*, and *gpia*—as well as fatty acid oxidation genes (*gcdh*, *asah1a*, *sdhb*, *sdhaf*2, *idh1*, *sdha*, *idh3a*) were significantly upregulated in SKLT-treated blastemas.

Interestingly, *aldh1a2*, encoding aldehyde dehydrogenase 1 family member A2, was downregulated in SKLT-treated fish. These molecular signatures are consistent with the reduced regenerative outgrowth observed at later stages in SKLT-treated animals (**Figure 4**).

### SKLT protects against RANKL-induced bone loss in a medaka model of osteoporosis

Whether the immunomodulatory properties of SKLT could prevent bone loss in a disease setting was investigated in the inducible osteoporosis medaka model Tg(*rankl*:HSE: CFP)^TG1135^, in which bone resorption is triggered by conditional overexpression of RANKL (19). Successful induction is monitored via cyan fluorescent protein (CFP) fluorescence, which is co-expressed with RANKL under the control of a bidirectional heat-shock promoter. Higher CFP intensity corresponds to greater RANKL expression and increased osteoclastic activity, resulting in bone loss.

**Figure 6:**
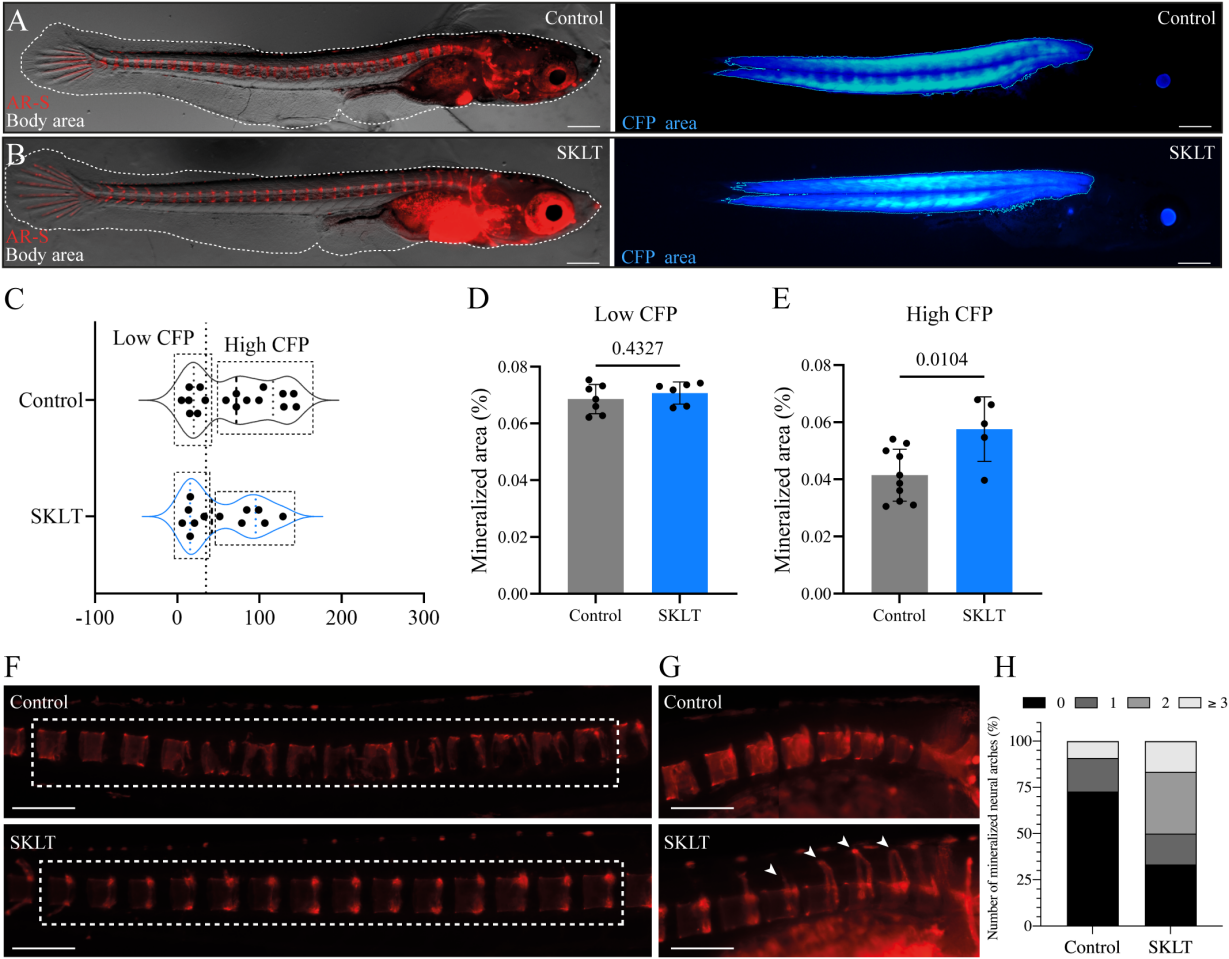
SKLT protects against bone loss in a medaka model of RANKL-induced osteoporosis. Mineralization of the vertebral column was assessed in adult medaka induced for osteoporosis and exposed to the ethanolic fraction of *S. costatum* (SKLT) or 0.1% ethanol (control). **(A, B)** Representative images illustrating the total body area (dotted line) and rankl-CFP*^+^* area in SKLT-treated **(A)** and control **(B)** fish. **(C)** Frequency distribution of the CFP mean intensity. **(D, E)** Quantification of the mineralized area of the vertebral column in the different fish clusters. **(F)** Representative images depicting alizarin red stained vertebral columns of control (upper image) and SKLT-exposed (lower image) fish in the High CFP intensity group. **(G)** Representative images depicting the number of mineralized neural arches in control (upper image) and SKLT-exposed (lower image) fish in the High CFP intensity group. **(H)** Number of mineralized neural arches in SKLT-exposed and control fish in the High CFP intensity group. Statistical differences were tested through an unpaired *t* test (*p* < 0.05). Scale bars are 360 µm in **(A, B)** and 730 µm in **(J, K)**.

To evaluate the protective effects of SKLT, medaka larvae were heat-shocked and then exposed to SKLT or vehicle for 6 days. RANKL expression levels were estimated by measuring CFP fluorescence intensity (**Figures 6A, B**). Because the distribution of CFP fluorescence intensity was bimodal or right-skewed, larvae were stratified into two subgroups based on CFP intensity (**Figure 6C**). Bone loss was assessed by measuring the mineralized area of the vertebral column following alizarin red staining. In the subgroup with low CFP fluorescence—indicative of low RANKL expression—no difference in vertebral mineralization was observed between control and SKLT-treated fish (**Figure 6D**). However, in fish with high CFP (and thus high RANKL) expression, exposure to SKLT significantly increased vertebral mineralization (**Figure 6E**).

Additionally, these fish exhibited a greater number of mineralized neural arches (**Figures 6F–H**). These findings suggest that SKLT provides protection against RANKL-induced bone loss in cases of strong RANKL induction, but has no effect when RANKL expression is low. Thus, SKLT appears to counteract the osteoporotic phenotype in a severity-dependent manner.

### SKLT inhibits osteoclastic differentiation in murine macrophages without inducing cytotoxicity

**Figure 7:**
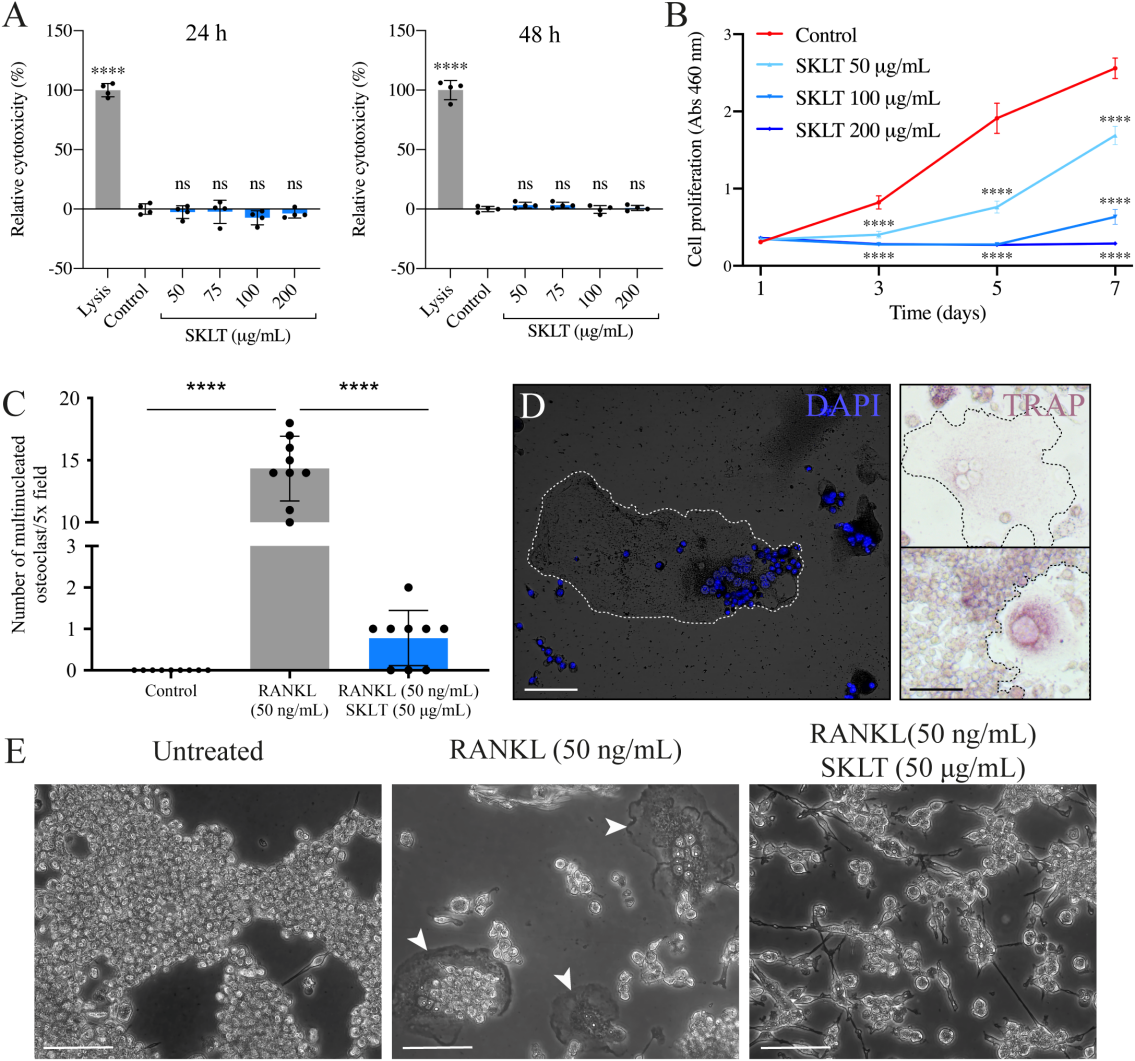
SKLT inhibits cell proliferation and osteoclastic differentiation in murine macrophages. The cytotoxicity, proliferation and osteoclastic differentiation of RAW 264.7 cells were assessed upon exposure to the ethanolic fraction of *S. costatum* (SKLT) or 0.1% ethanol (control). **(A)** Cytotoxicity (LDH assay) in cells exposed for 24 h (left) or 48 h (right) to different concentrations of SKLT. **(B)** Proliferation (XTT assay) of cells exposed to different concentrations of SKLT. **(C)** Number of multinucleated osteoclasts per field (magnification 5.0X). **(D)** Representative fluorescence image of DAPI-stained multinucleated osteoclasts (left) and brightfield images of TRAP^+^ osteoclasts (right). **(E)** Representative phase-contrast images of the 3 experimental groups. Arrowheads indicate multinucleated osteoclasts. Differences between each treatment and the control were tested via one-way ANOVA followed by Dunnett’s multiple comparison test (*p* < 0.05). Asterisks indicate values significantly different at *p* < 0.0001 (****). Scale bars are 150 µm.

The ability of SKLT to inhibit osteoclast differentiation was evaluated using the murine macrophage cell line RAW 264.7, which undergoes osteoclastic differentiation upon exposure to RANKL. To assess the effects of SKLT on cell viability and proliferation, RAW 264.7 cells were exposed to various concentrations of SKLT (50, 100, and 200 µg/mL) for 24 and 48 hours. No cytotoxic effects were observed at any concentration (**Figure 7A**). However, significant inhibition of cell proliferation was detected after 1, 3, 5, and 7 days of treatment, even at the lowest concentration (50 µg/mL) (**Figure 7B**). To evaluate osteoclastogenesis, cells were co-treated for 6 days with RANKL (50 ng/mL) and SKLT (50 µg/mL), and stained with DAPI and TRAP (**Figures 7C, D**). No large multinucleated (>2 nuclei) TRAP^+^ cells were detected in the negative control group (no RANKL), while the RANKL-treated group produced an average of 14.3 ± 2.6 osteoclasts per 5.0x field. Co-treatment with SKLT significantly suppressed osteoclast differentiation, reducing the number of multinucleated TRAP^+^ osteoclasts to 0.78 ± 0.67 per 5.0x field (**Figures 7C, E**).

These results confirm that SKLT exerts anti-proliferative and anti-osteoclastogenic effects on murine macrophages without causing cytotoxicity, supporting its cross-species activity and potential as an immunomodulatory inhibitor of osteoclast formation.

## Discussion

The immune system is increasingly recognized as a key driver of bone erosive pathologies, including primary osteoporosis, where dysregulation of innate and adaptive immune cells contributes to sustained bone resorption (30, 51).

Although no currently available therapeutics act via immunomodulation, growing evidence supporting immune involvement in bone homeostasis has laid the foundation for novel therapeutic approaches targeting immune pathways (52, 53).

Here, the immunomodulatory potential of an ethanol-soluble fraction from the marine microalga *Skeletonema costatum* in treating bone loss was explored. In a zebrafish fin regeneration model (45), exposure to this fraction (SKLT) led to reduced ray bifurcation. During fin regeneration, the exact positioning of the bifurcation point in regenerating rays depends on the conjunct activity of re-differentiated osteoblasts (51, 54, 55), and osteolytic tubules (OLTs) (48). A shift in branching position indicates that SKLT modulates the morphogenetic pattern of ray formation, potentially by altering the balance between osteogenic and osteoclastic activity, as previously observed for other osteo-modulatory drugs (48). Previous work has demonstrated that osteoanabolic and antiresorptive agents alter ray bifurcation patterns, making this model a suitable *in vivo* screening platform for compounds that affect bone remodeling (48). Therefore, the distalization of ray bifurcations in SKLT-treated fins reflects either enhanced osteogenesis or suppressed resorption.

Given prior reports of anti-inflammatory (42, 43), and pro-mineralogenic (44) activities in *S. costatum* extracts, and the central role of inflammation in osteoclastogenesis (16), we hypothesized that SKLT impairs osteoclast function through immunomodulation.

This was supported by reduced expression of cathepsin K (an osteoclast precursor marker) and weaker TRAP activity (a marker of mature osteoclasts), consistent with previous findings (48).

These data suggest that SKLT delays osteoclast precursor recruitment or differentiation during early fin regeneration. Interestingly, SKLT also impaired late-stage regenerative outgrowth.

Since inflammation is essential for initiating regeneration across multiple tissues—including fin (51, 55), heart (56, 57), and spinal cord (58)—its suppression could have impaired tissue regeneration (59).

This hypothesis is supported by studies showing that pharmacologically suppressing inflammation in zebrafish leads to impaired fin regeneration (60, 61). Similarly, macrophage ablation in adult zebrafish reduces regeneration and abolishes ray bifurcation (62).

Transcriptomic data confirmed that SKLT downregulates genes associated with early inflammatory responses in blastema tissue, including *il20ra*, a receptor for the pro-inflammatory and pro-osteoclastogenic cytokine in interleukin 20 (IL-20) (63, 64).

SKLT also affected adaptive immune signaling. Downregulation of genes involved in T-cell activation, interferon gamma (IFNγ) signaling, and antigen presentation was observed in fish treated with SKLT. The contribution of T-cells to estrogen deficiency-induced bone loss has been known since the early 2000s (65), when the field of osteoimmunology was formally proposed (66). Numerous T-cell–related genes were suppressed in SKLT-treated fish, including *cd4*, *cd74a/b*, *ccl19a.1*, and *jak1*, all of which are associated with osteoclastogenesis (30). Similarly, components of the interferon pathway (*ifng1r*, *irf4b*, *irf10*) *(* 67–69), and *itk*, encoding the IL2-inducible T-cell kinase, a regulator of natural killer T-cells (NKT) differentiation (70), were downregulated.

Notably, *il7ra*, linked to pro-osteoclastogenic cytokine production (71), and bone resorption–formation uncoupling (72), was also suppressed.

In contrast, *il13ra2*, encoding a receptor for the osteoprotective cytokine interleukin 13 (IL-13) (73, 74), was upregulated, suggesting a shift toward anti-inflammatory signaling.

Modulation of genes involved in osteoimmune paracrine communication was also observed. In medaka, Cxcl9l, produced by osteoblasts under osteoporotic conditions, directs a subset of macrophages toward osteoclast fate via Cxcr3.2 (75).

In zebrafish, *cxcr3.1*, one of the three Cxcr3 paralogs, was downregulated, possibly indicating reduced responsiveness of osteoclast progenitors to chemotactic signals. Moreover, the matrix metalloproteinase *mmp13b*, recently implicated in osteoclast maturation and resorptive function (76), was also downregulated in SKLT-exposed fish.

Together, these data indicate that SKLT induces broad suppression of immune-related molecular programs at 24 hpa, including those regulating T-cell activation, antigen presentation, and macrophage–osteoblast cross-talk. These immunological shifts correlate with reduced recruitment and maturation of osteoclast precursors *in vivo*.

Additionally, transcriptomic data indicate that SKLT reduced the expression of *aldh1a2*, the main enzyme responsible for the synthesis of retinoic acid (RA), a molecule whose expression is normally elevated in early stages of fin regeneration (77–79). Given RA’s role in promoting blastema proliferation and growth, and in coordinating osteoblast dedifferentiation, proliferation, and redifferentiation during fin regeneration (77–79), its suppression by SKLT likely contributes to the observed downregulation of cell cycle genes, and is consistent with our *in vitro* data showing an anti-proliferative effect on murine macrophages.

Subsequently, the potential of SKLT to prevent bone loss in a disease context was validated in a medaka model of RANKL-induced osteoporosis (19), which showed prevention of bone loss, consistent with the suppression of osteoclast activation found in zebrafish and by our transcriptomic analysis. These effects were reproduced in a murine macrophage line, where SKLT reduced the formation of multinucleated osteoclasts under RANKL stimulation, supporting its cross-species activity and translational potential (80–82).

Taken together, our findings establish SKLT as a source of immunomodulatory and anti-osteoclastogenic activity, and based on the known phytochemical repertoire of *Skeletonema costatum*, including carotenoids such as zeaxanthin (83), long-chain polyunsaturated fatty acids like palmitoleic acid, and phenolic derivatives (83, 84). These classes of compounds emerge as plausible mediators of the observed anti-osteoclastogenic effects, as previous works have shown (85–88), warranting future bioassay-guided fractionation to pinpoint their contribution to bone-protective activity.

## Conclusions

The findings presented here highlight the therapeutic potential of targeting both innate and adaptive immune responses to treat bone erosive pathologies. We demonstrate that *Skeletonema costatum* contains ethanol-soluble immunomodulatory compounds capable of downregulating T-cell activation, antigen presentation, and macrophage differentiation toward the osteoclastic lineage. Although the precise identity of the active component(s) remains to be determined and represents a limitation of the present study, our data establish a foundation for future pharmacological exploration of this microalga. While further work is needed to isolate and characterize individual bioactives, the consistent immunomodulatory and anti-osteoclastogenic activity observed across zebrafish, medaka, and murine models supports the broader therapeutic promise of this ethanol-soluble fraction.

These results align with growing calls for holistic, nutraceutical-based approaches to managing chronic diseases involving immune dysregulation and bone loss.

Our study provides a proof of concept for the use of marine-derived immunomodulators to prevent bone erosion by regulating immune-driven osteoclastogenesis.

## Materials and methods

### Preparation of microalgal ethanolic fractions

The freeze-dried biomass of *Skeletonema costatum* (Necton S.A., Olhão, Portugal) was macerated with 96% ethanol (Laborspirit Lda, Lisbon, Portugal) at a biomass–solvent ratio of 1 g:40 mL (M/V) by gently stirring at 24 °C for 18 h. The macerate was centrifuged for 5 min at 1,000 × g via an Allegra 6R centrifuge (Beckman Coulter Inc., Brea, USA), and the supernatant was collected. The pellet was washed twice with 96% ethanol, and all the supernatants were pooled and then vacuum filtered sequentially through 0.45 µm and 0.22 µm nylon membranes (Labbox Labware S.L., Barcelona, Spain). The filtrate was concentrated with an RV 10 digital rotary evaporator (IKA-Werke GmbH & Co. KG, Staufen im Breisgau, Germany), with the temperature set at 40 °C and pressure at 178 mbar, until a dense, paste-like extract was obtained. The extraction yield (37.9 ± 2.4%) was calculated from 2 mL aliquots (*n* = 3) placed under a gentle flow of 99.8% nitrogen until complete evaporation of the solvent.

### Fish maintenance

Zebrafish wild-type line AB and transgenic line Tg(Ola.*ctsk*:FRT-DsRed-FRT-Cre, *myl7*:EGFP)^mh201,48^, referred to as Tg(*ctsk*:DsRed) throughout the manuscript, were maintained in a water recirculating system ZebTEC (Tecniplast, Buguggiate, Italy) with the following conditions: temperature 28 ± 0.1 °C, pH 7.5 ± 0.1, conductivity 700 ± 50 μS, ammonia and nitrites at levels below 0.1 mg/L, nitrates lower than 50 mg/L, and a photoperiod of 14:10 h light-dark. The medaka transgenic line Tg(*rankl*:HSE: CFP)^TG1135^, hereafter referred to as Tg(*rankl*:HSE: CFP), was purchased from the National BioResource Project Medaka (NBRP Medaka) (89) and maintained in a water recirculating system with the following conditions: temperature 27 ± 0.1 °C, pH 7.0 ± 0.1, conductivity 300 ± 100 μS, ammonia and nitrites at levels below 0.1 mg/L, nitrates lower than 50 mg/L and a photoperiod of 14:10 h light–dark. For zebrafish and medaka, system water was prepared by supplementing reverse osmosis water with a salt mixture (Instant Ocean, City, USA) and sodium bicarbonate (Sigma–Aldrich, St. Louis, USA). All the fish were fed commercial dry food Zebrafeed (Sparos Lda, Olhão, Portugal) daily.

### Zebrafish caudal fin regeneration assay

The caudal fin of wild-type or transgenic adult zebrafish aged 3–4 months was amputated 1–2 segments anterior to the bifurcation of the most peripheral branching lepidotrichia, as described by Cardeira et al. (2016) (45). For the first experiment, AB wild type zebrafish were sampled at a single time-point, 5 days post-amputation (dpa). After finectomy, fish (*n* = 14 for SKLT, and *n* = 32 for CTRL) were placed at 33 ± 1 °C in 3 L plastic containers at a density of 5 fish/L. The fish were exposed to the *S. costatum* ethanolic fraction (SKLT) at 56 µg/mL or ethanol (CTRL) at 0.1% supplemented in system water for 5 days, to evaluate their mineralogenic performance.

For the second experiments to track osteoclastic cells, transgenic Tg(*ctsk*:DsRed) fish (*n* = 18) were amputated and then exposed to 56 µg/mL (SKLT) or 0.1% ethanol (CTRL).

In all experiments, moderate water dynamics and air-water exchanges were facilitated by bubbling the fish tanks with an air pump. Water quality parameters were monitored daily and maintained stable for the duration of the experiment as follows: dissolved oxygen, 7.0 ± 0.5 mg/L; pH, 7.4 ± 0.2; and conductivity, 680 ± 20 μS. Wild-type fish were sacrificed at 120 hours post-amputation (hpa) via lethal anesthesia with 0.6 mM tricaine methanesulfonate (MS-222, pH 7.0; Sigma–Aldrich), immersed for 30 min in 0.03% alizarin red S (AR-S, pH 7.4; Sigma–Aldrich) and washed two times for 5 min with system water. The stained fish were imaged for fin morphometric analysis. Transgenic For the osteoclast-tracking experiment, At 24 hours post-amputation, half of the transgenic Tg(*ctsk*:DsRed) (*n* = 9) for each group were given a lethal anesthesia with MS-222, imaged for *ctsk* signal, then caudal fins were dissected and collected for TRAP staining. The other half (*n* = 9) were continuously kept in 56 µg/mL (SKLT) or 0.1% ethanol (CTRL), and fluorescence was at different time points (48, 72, 96, 120 and 240 hpa). Fish were immersed for 30 min in 0.2% calcein (pH 7.4; Fluorexon, Sigma–Aldrich), and washed twice in system water for 10 min. After staining, the fish were anesthetized for 5 min in tricaine and imaged.

For bulk the RNA sequencing (RNA-seq) analysis, fin blastemas were collected at 24 hpa, pooled (*n* = 5, 10 blastemas per pool), and stored at -80 °C until further processing.

### TRAP activity in caudal fins

Caudal fins were amputated at the level of the caudal peduncle, washed once with 1X phosphate-buffered saline (PBS, pH 7.4) and fixed for 4 h in 4% paraformaldehyde solution (PFA, solubilized in PBS, pH 7.4) at 24 °C. Tartrate-resistant acid phosphatase (TRAP) staining was performed as previously described by Blum & Begemann (2015) (78), and the fins were imaged as described below.

### Morphometric analysis of caudal fins

Fins were imaged under an MZ10F fluorescence stereomicroscope (Leica, Wetzlar, Germany) coupled to a DFC7000T color camera (Leica). Bright-field images were collected to assess the progression of fin regeneration. Fluorescence images were collected to assess (i) *de novo* bone formation in wild-type fish stained with AR-S or calcein and (ii) the involvement of *ctsk*-expressing cells in transgenic fish labeled with DsRed. Bright-field images were acquired with an exposure time of 1 ms. Fluorescence images were acquired with the filter set ET560/40x - ET630/75m and an exposure time of 600 ms for mCherry, and the filter set ET470/40x - ET525/50m and an exposure time of 80 ms for GFP. Other image parameters were as follows: gamma, 1.00; image format, 1920×1440 pixels; and binning, 1×1. The fluorescence images were analyzed using ImageJ software version 2.0.0-rc-69/1.52p and processed with the ZFBONE toolset for caudal fin morphometrics (90). Fin regeneration and mineralization were assessed following the method described by Cardeira et al. (2016) (45) by calculating the regenerated area (REG), the stump width (STU), the mineralized area (MIN), and the average width of the rays before the amputation (RAYs). Fin ray patterning was also assessed by calculating the average ray width ratio ( $\bar{W}$ ) and the average bifurcation ratio ( $\bar{Bt}$ ), as shown in **Supplementary Equations a, b**. For the quantification of the *ctsk* signal in transgenic fish, the DsRed-positive area was measured using a color threshold on fluorescence images and normalized with REG/STU. To quantify the TRAP signal, TRAP-positive areas were measured using a color threshold on bright-field images and subsequently normalized with REG/STU.

### RNA preparation

Total RNA was extracted from pools of blastemas at 24 hpa (*n* = 5) using NZYol (NZYTech, Lisbon, Portugal) and quantified using a NanoDrop OneC spectrophotometer (Thermo Fisher Scientific, Waltham, USA). The RNA integrity was confirmed using an Experion Automated Electrophoresis system (Bio-Rad, Hercules, USA). Only RNA with an RNA integrity number (RIN) greater than 7 was used.

### Bulk RNA sequencing and analysis of differentially expressed genes

Bulk RNA sequencing was outsourced to STABVIDA Lda (Caparica, Portugal). DNA libraries were constructed using a Stranded mRNA Library Preparation Kit (STABVIDA) and sequenced on a NovaSeq platform (Illumina, San Diego, USA) to generate 150 bp paired-end sequencing reads. The raw sequence data were processed using CLC Genomics Workbench 12.0.3. Trimming was performed in 3 steps: quality trimming on the basis of quality scores (error probability of 0.01), ambiguity trimming (ambiguous limit of 2 nucleotides), and length trimming to discard reads shorter than 30 nucleotides. The quality-checked sequencing reads were mapped against the zebrafish reference genome GRCz11 (GCF_000002035.6) using length fraction and similarity fraction equal to 0.8. The TPM (transcripts per million) and RPKM (reads per kilobase of transcript per million mapped reads) values were then determined from the mapped data. Differential expression analysis was performed with the multifactorial EdgeR method in R. Overall, the gene expression results were represented with a clustering heatmap of gene expression, with a principal component analysis (PCA) plotting the amount of variance explained by the two principal components and a volcano plot representing overall gene expression with a fold change on the x-axis and significance of expression on the y-axis (**Supplementary Figure S2**). These overall representations of gene expression were performed to assess variation patterns in the gene expression dataset and identify outlier samples for quality control. For functional annotation enrichment analysis, the lists of upregulated and downregulated genes were analyzed with the online resource Database for Annotation, Visualization and Integrated Discovery (DAVID) (91, 92) for Gene Ontology–Biological Processes (GO: BP) with FDR< 0.01 and enriched biological processes.

### Medaka model of RANKL overexpression-induced osteoporosis

Eggs of the medaka line Tg(*rankl*:HSE: CFP) were produced following an in-house breeding program and maintained in Petri dishes with 40 mL of system water supplemented with 0.0002% (w/v) methylene blue until 8 days post-fertilization (dpf). At 8 dpf, the hatched larvae were incubated at 39 °C for 2 h (heat shock) and screened for CFP (cyan fluorescent protein) signal using a fluorescence microscopy (see parameters below). CFP-positive fish were distributed into a 6-well plate at a density of 5 larvae/well. Each well was filled with 10 mL of system water supplemented with SKLT at 56 µg/mL or 0.1% ethanol (vehicle). The treatment was renewed 100% daily. Six days after heat shock, the larvae (*n* = 17 for CTRL, and *n* = 12 for SKLT) were sacrificed with a lethal dose of tricaine (see above), stained with AR-S and imaged as described above. Bright-field and fluorescence images were collected for each fish. Bright-field images were acquired with an exposure time of 2 ms. AR-S fluorescence images were acquired using the filter set ET560/40x - ET630/75m and an exposure time of 700 ms. CFP fluorescence images were acquired using the filter set ET436/20x - ET480/40m and an exposure time of 200 ms. Other image parameters were as follows: gamma 1.00, image format 1920×1440 pixels, and binning 1×1. Images were analyzed using ImageJ using two *ad hoc* macros (**Supplementary Files 1**, **2**). CFP fluorescence images were transformed into 8-bit images, and the CFP-positive area was measured using a color threshold (min intensity of 7 and max intensity of 255). The pixel mean intensity inside the CFP-positive area was calculated and used as a proxy for CPF fluorescence intensity. AR-S fluorescence images were transformed into 8-bit images, and AR-S positive areas were measured using a color threshold (min intensity 5 and max intensity 255). The AR-S positive area was normalized using the total body area (determined manually from bright-field images) to correct for inter-specimen size variation. The AR-S positive area in the abdominal and caudal vertebrae was used as a proxy for mineralization of the vertebral column. The nomenclature proposed by Di Biagio et al. (2022) (83) was used to identify vertebrae from 4-29. The number of mineralized neural arches was manually counted in each fish from the AR-S images. Following the collection of the data, the frequency distribution of the CFP intensity and tested data normality was determined through an Anderson–Darling test (p< 0.05). Normality was not met in the control group (p = 0.0035), but CFP intensity was normally distributed in SKLT-exposed fish (p = 0.220). The data distribution for CFP intensity was right skewed (median< mean) for the control group and bimodal for the SKLT group. These findings implied that clustering was necessary to proceed with the statistical analysis. The fish were therefore grouped according to CFP intensity into “High CFP” (mean pixel intensity > 50) and “Low CFP” (mean pixel intensity< 50) for further analysis.

### Culture of mouse RAW 264.7 macrophages

RAW 264.7 cells were cultured in 10 cm cell culture dishes with Dulbecco’s Modified Eagle Medium (DMEM) supplemented with 10% fetal bovine serum (FBS; Sigma–Aldrich), 1% of penicillin–streptomycin (100x), 1% of 200 mM L-glutamine, and 0.2% of 250 µg/mL amphotericin B at 37 °C in a humidified atmosphere containing 5% CO_2_. PreconfluenT-cell cultures were sub-cultured at a ratio of 1:4 every other day using trypsin-EDTA solution (0.2% trypsin, 1.1 mM EDTA: prepared in 1x PBS, pH 7.4). All cell culture reagents were obtained from GIBCO-Thermo Fisher Scientific, unless otherwise stated.

### Cytotoxicity and cell proliferation

An LDH cytotoxicity assay kit and a XTT-cell proliferation assay kit (Canvax Biotech, Córdoba, Spain) were used to evaluate the effects of SKLT on cellular toxicity and proliferation, respectively. For cytotoxicity, RAW 264.7 cells were seeded in a 96-well plate at 1.0 × 10^4^ cells/well (*n* = 4) in 100 µL of culture medium supplemented with either the SKLT at 50, 75, 100, 200 µg/mL or 0.1% ethanol (vehicle) and cultured for 24 and 48 h. Relative cytotoxicity was calculated as a percentage of LDH lysis control. For cell proliferation, RAW 264.7 cells were seeded in a 96-well plate at 500 cells/well (*n* = 6) in 100 µL of culture medium supplemented with either the SKLT at 50, 75, 100, 200 µg/mL or vehicle and cultured for 1, 3, 5 or 7 days. The culture medium was renewed every other day. Relative cell proliferation was calculated as a percentage of the negative control.

### Osteoclast differentiation

RAW 264.7 cells were seeded in 12-well plates at 1.5 × 10^4^ cells/well in 1 mL of culture medium (as described above, *n* = 9) supplemented with either SKLT at 50 µg/ml or 0.1% ethanol (vehicle) and treated for 6 days with 50 ng/mL RANKL (Preprotech, London, UK) prepared in 0.1% bovine serum albumin (BSA) to induce their differentiation into multinucleated osteoclasts. The experimental conditions were as follows: undifferentiated control (0.1% ethanol); differentiated control (50 ng/mL RANKL + 0.1% ethanol); differentiated cells exposed to SKLT (50 ng/mL RANKL + 50 µg/mL SKLT in 0.1% ethanol). The culture medium was freshly prepared and replaced daily. Osteoclast differentiation was assessed by counting the number of tartrate-resistant acid phosphatase (TRAP)-positive cells and the number of nuclei following 4′,6-diamidino-2-phenylindole (DAPI) staining. For this purpose, the cells were fixed in 4% PFA at 24 °C (RT) for 10 min and stained according to the protocol of Blum & Begemann (2015) (78). The cells were subsequently stained with DAPI for nuclear detection and counting. The cells were imaged using an Axio Vert. A1 inverted microscope (ZEISS, Jena, Germany) coupled with (i) an Axiocam 202 monocolor camera (ZEISS) for DAPI images, or (ii) a VWR VisiCam 5 Plus (VWR, Radnor, USA) for bright-field TRAP images. The number of multinucleated osteoclasts per field (5.0X magnification; *n* = 9) was calculated considering only TRAP+ cells with at least two nuclei.

### Statistical analysis

For all the experiments, normality was tested with a D’Agostino–Pearson omnibus normality test or with an Anderson–Darling test (p< 0.05). Homoscedasticity was tested through the Brown–Forsythe test (p< 0.05). When the distribution of the data of all the experimental groups resulted in normal and homogeneous, statistical differences between the control and the treated groups were tested with either an unpaired t test or one-way ANOVA, followed by Dunnett’s multiple comparison test (p< 0.05). If the distribution of the data of any of the experimental conditions resulted in non-normal or nonhomogeneous, significant differences between the control and the treatments were tested with a Mann–Whitney test or a nonparametric test followed by Dunn’s multiple comparison test (p< 0.05). Statistical analyses were performed using Prism version 9.00 (GraphPad Software Inc., La Jolla, United States).

## Data Availability

The datasets presented in this study can be found in online repositories. The names of the repository/repositories and accession number(s) can be found below:https://www.ebi.ac.uk/biostudies/, RNA sequencing data have been uploaded onto the free repository BioStudies EMBL-EBI with accession number E-MTAB-14677, and will be made publicly available upon article acceptance. All other raw data generated in this study will be made available upon request.
