# Supplementary material for: Immunomodulatory inhibition of osteoclastogenesis by a marine microalgal ethanol fraction targeting T-cells, antigen presentation, and macrophage fate
Source: Front Immunol. 2025 Oct 10;16:1655321. doi: 10.3389/fimmu.2025.1655321 (PMC12549262; doi:10.3389/fimmu.2025.1655321)
Supplement: Supplementary file 1 [file DataSheet1.docx]

Supplementary Material

Equations

***(a)*** Ray width ratio (*W*) = $\frac{\sum_{1}^{n} w1, w2,wn}{n}$*,* Average width ratio ($\overline{W}$) = $\frac{\sum_{1}^{M} Wm}{RAYs}$ where,

*w* = width of the segments after the amputation

*n* = number of segments after the amputation

*RAYs* = average width of the first segment before the amputation

*m* = number of rays

(***b***) Ray bifurcation ratio (*Bt*) = *BIF/TOT*, Average width ratio ($\overline{Bt}$) = $\frac{\sum_{1}^{M} Bt1, Bt2, Btm}{m}$ where,

*BIF* = bifurcation length

*TOT* = regenerated ray length

*m* = numbers of rays

## Supplementary Figures

**
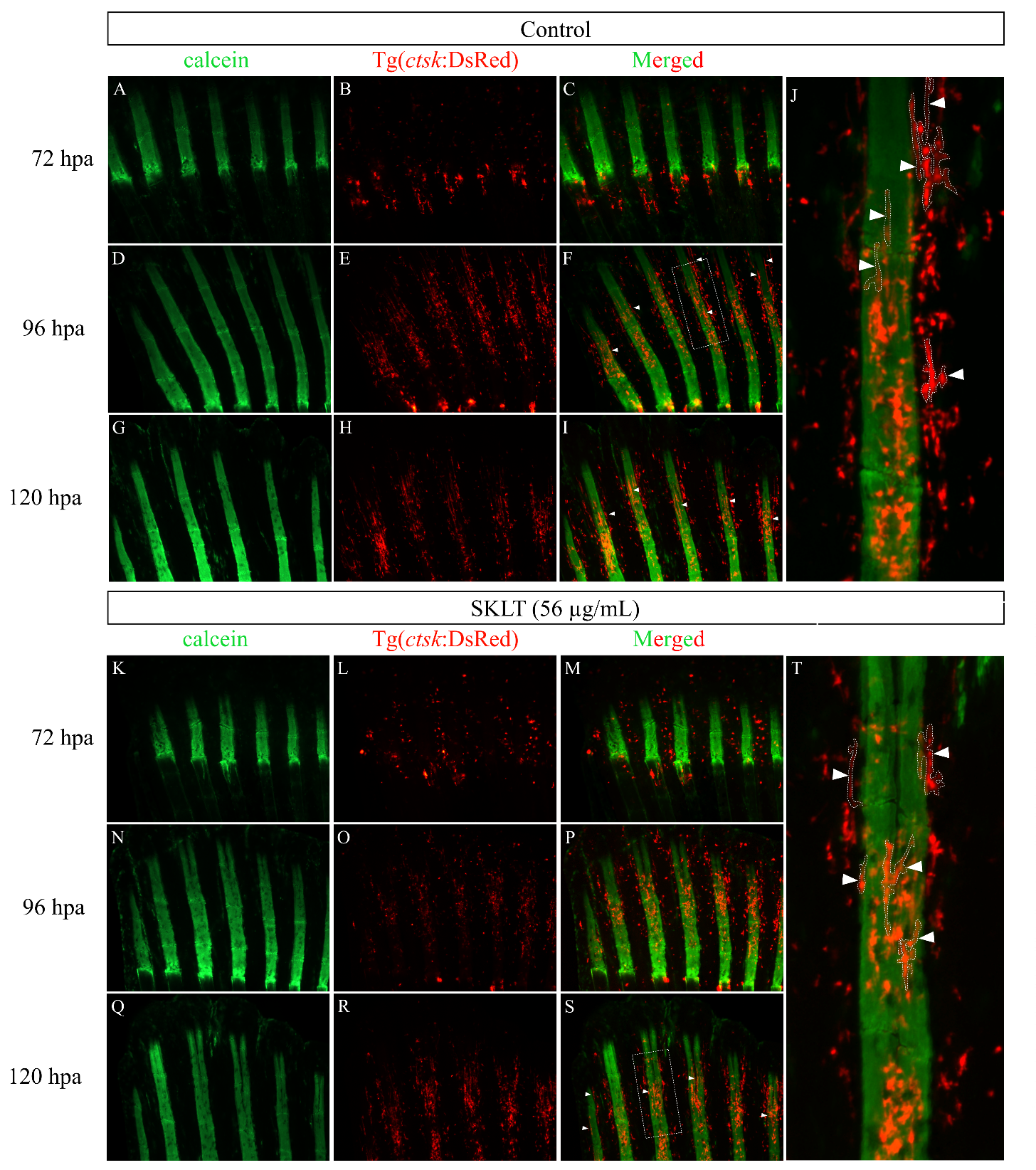
**

**Figure S1. The ethanolic extract of *Skeletonema costatum* does not prevent the appearance of *ctsk^+^* OLTs at later stages of fin regeneration.** Presence of tubular and elongated *ctsk^+^* osteolytic tubules in the regenerating caudal fin of adult zebrafish exposed to 0.1% ethanol (Control; **A-J**) or to the ethanolic extract of *Skeletonema costatum* at the concentration of 56 µg/mL (SKLT; **K-T**). Osteolytic tubules (OLTs) are observed in most fins at 96 hpa in control fish (**J**), and at 120 hpa (**T**) in SKLT-treated fish. hpa, hours post amputation; white arrowheads, ctsk+ osteolytic tubules associated with the regenerated bony rays.

**
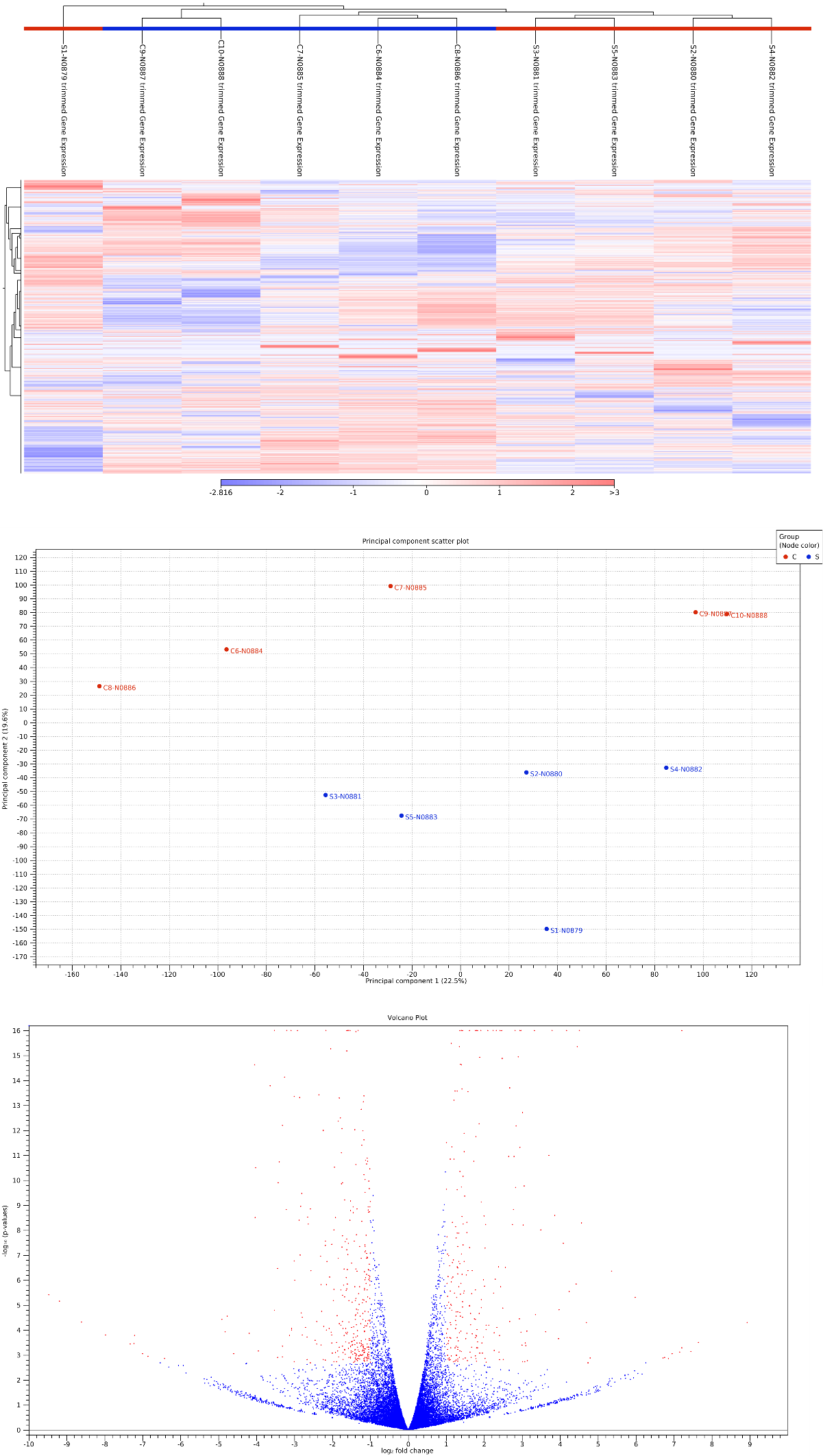
**

**Figure S2. Overall gene expression results from RNAseq analysis of SKLT-exposed vs control specimens.**

(**A**) Heatmap of the expression analysis created with the results of the gene expression. The red colour represents over-expression, while the blue colour is under-expression. (**B**) PCA corresponds to the gene expression of the groups of samples. The first principal component is shown on the X-axis, and the second principal component is shown on the Y-axis. The value after the principal component identifier displays the amount of variance explained by this principal component. (**C**) Volcano plot representing overall gene expression with fold change in the x-axis and significance of expression in the y-axis, with significantly differentially expressed genes represented in red dots and not significantly differentially expressed genes in blue dots.

Supplementary Data

Macro scripts for Fiji cited in the manuscript

**Supplementary macro 1**

/*

DEVELOPER: Marco Tarasco

EMAIL: marcotar90@gmail.com

*/

//Macro developed for the analysis of body area in transgenic medaka.

macro "Measure area Action Tool - C000D06D09D15D1aD25D2aD34D37D38D3bD44D4bD53D54D5bD5cD63D6cD73D76D79D7cD83D87D88D8cD93D9cDa2DadDb2DbdDc1DceDd1Dd2Dd3DdcDddDdeDe0De1De4DebDeeDefDf4Dfb"{

//Menu options

Dialog.create("Measure area");

Dialog.addRadioButtonGroup("Mode", newArray("Single","Batch"),1, 2, "Single");

Dialog.show();

mode=Dialog.getRadioButton();

// analyse single image or batch?

if (mode=="Single") {

doAnalysis();

}

else {

dir=getDirectory("Choose a Directory");

list=getFileList(dir);

for (i = 0; i < list.length; i++) {

if (endsWith(toLowerCase(list[i]), ".bmp")||endsWith(toLowerCase(list[i]), ".tif")||endsWith(toLowerCase(list[i]), ".jpg")){

if(!File.exists(dir+"analyzed/"+list[i]+"_analyzed.jpg")){

open(dir+list[i]);

doAnalysis();

run("Close All");

}

}

}

}

function doAnalysis(){

if (nImages==0) waitForUser("Please open an image");

if (nImages==0) exit("No image was opened!!");

run("Set Scale...", "distance=0 known=0 pixel=1 unit=pixel");

roiManager("reset");

roiManager("Show None");

dir=getDirectory("image");

img=getTitle();

//measure fish area

setTool("polygon");

while(selectionType==-1){

waitForUser("Select "+"Area");

}

roiManager("Add");

List.setMeasurements;

areaFish=List.getValue("Area");

setResult("Label", nResults, img);

setResult("Fish area", nResults-1, areaFish);

roiManager("Select", 0);

roiManager("Set Color", "magenta");

roiManager("Rename", "Fish area");

roiManager("Deselect");

roiManager("show all without labels");

run("Flatten");

getDateAndTime(year, month, dayOfWeek, dayOfMonth, hour, minute, second, msec);

dateTag=""+year+"-"+IJ.pad((month+1), 2)+"-"+IJ.pad((dayOfMonth), 2)+"_";

direct=dir+"analyzed/";

File.makeDirectory(direct);

roiManager("Save",direct+img+"_RoiSet.zip");

saveAs("jpeg",direct+img+"_analyzed");

Table.save(direct+dateTag+"_Results.xls");

run("Close All");

}

}

**Supplementary macro 2**

/*

DEVELOPER: Marco Tarasco

EMAIL: marcotar90@gmail.com

*/

//Macro developed for the analysis of mineralized area in transgenic medaka.

macro "Measure fluorescence Action Tool - C000D06D09D15D1aD25D2aD34D37D38D3bD44D4bD53D54D5bD5cD63D6cD73D76D79D7cD83D87D88D8cD93D9cDa2DadDb2DbdDc1DceDd1Dd2Dd3DdcDddDdeDe0De1De4DebDeeDefDf4Dfb"{

//Menu options

Dialog.create("Measure fluorescence");

Dialog.addRadioButtonGroup("Mode", newArray("Single","Batch"),1, 2, "Single");

Dialog.show();

mode=Dialog.getRadioButton();

// analyse single image or batch?

if (mode=="Single") {

doAnalysis();

}

else {

dir=getDirectory("Choose a Directory");

list=getFileList(dir);

for (i = 0; i < list.length; i++) {

if (endsWith(toLowerCase(list[i]), ".bmp")||endsWith(toLowerCase(list[i]), ".tif")||endsWith(toLowerCase(list[i]), ".jpg")){

if(!File.exists(dir+"analyzed/"+list[i]+"_analyzed.jpg")){

open(dir+list[i]);

doAnalysis();

run("Close All");

}

}

}

}

function doAnalysis(){

if (nImages==0) waitForUser("Please open an image");

if (nImages==0) exit("No image was opened!!");

roiManager("reset");

roiManager("Show None");

dir=getDirectory("image");

img=getTitle();

run("Set Scale...", "distance=0 known=0 pixel=1 unit=pixel");

//measure fish area

setTool("polygon");

while(selectionType==-1){

waitForUser("Select "+"Area");

}

run("8-bit");

roiManager("Add");

List.setMeasurements;

areaFish=List.getValue("Area");

thr1Ch=colorThresholding1Ch();

run("Restore Selection");

// FluoArea=List.getValue("RawIntDen")/255;

run("Create Selection");

roiManager("add");

close("*");

open(dir+img);

run("Set Scale...", "distance=0 known=0 pixel=1 unit=pixel");

//Rename and color ROIs

roiManager("Select", 0);

roiManager("Set Color", "magenta");

roiManager("Rename", "Roi area");

roiManager("select", newArray(0,1));

roiManager("AND");

roiManager("add");

roiManager("Select", 2);

roiManager("Set Color", "red");

roiManager("Rename", "Fluorescent area");

List.setMeasurements;

getRawStatistics(nPixels, mean, min, max, std, histogram);

FluoArea=List.getValue("Area");

TOtIntensity=nPixels*mean;

MeanIntensity=mean;

roiManager("deselect");

roiManager("select", 1);

roiManager("delete");

setResult("Label", nResults, img);

setResult("ROI area", nResults-1, areaFish);

setResult("Signal area", nResults-1, FluoArea);

setResult("Signal area Tot. int.", nResults-1, TOtIntensity);

setResult("Signal area Mean int.", nResults-1, MeanIntensity);

setResult("MIN Thr", nResults-1, thr1Ch[0]);

setResult("MAX Thr", nResults-1, thr1Ch[1]);

roiManager("Deselect");

roiManager("show all without labels");

run("Flatten");

getDateAndTime(year, month, dayOfWeek, dayOfMonth, hour, minute, second, msec);

dateTag=""+year+"-"+IJ.pad((month+1), 2)+"-"+IJ.pad((dayOfMonth), 2)+"_";

direct=dir+"analyzed/";

File.makeDirectory(direct);

roiManager("Save",direct+img+"_RoiSet.zip");

saveAs("jpeg",direct+img+"_analyzed");

Table.save(direct+dateTag+"_Results.xls");

run("Close All");

}

//------------------Threshold functions---------------------------//

function colorThresholding1Ch(){

run("Enhance Contrast", "saturated=0.35");

setAutoThreshold("IJ_IsoData dark");

run("Threshold...");

setOption("BlackBackground", false);

waitForUser("Adjust threshold then press ok");

getThreshold(lower, upper);

run("Convert to Mask");

return newArray(lower,upper);

}

}
